# Near Infrared–Circularly Polarized Luminescence/Circular Dichroism Active Yb(III) Complexes Bearing Both Central and Axial Chirality

**DOI:** 10.1021/acs.inorgchem.4c05420

**Published:** 2025-03-13

**Authors:** Silvia Ruggieri, Oliver George Willis, Silvia Mizzoni, Enrico Cavalli, Martina Sanadar, Andrea Melchior, Francesco Zinna, Lorenzo Di Bari, Giorgiana Denisa Bisag, Mariafrancesca Fochi, Luca Bernardi, Fabio Piccinelli

**Affiliations:** a Luminescent Materials Laboratory, DB, University of Verona, and INSTM, UdR Verona, Verona 37134, Italy; b Department of Chemistry and Industrial Chemistry, University of Pisa, Pisa 56124, Italy; c Department of Chemistry, Life Sciences and Environmental Sustainability, Parma University, Parma 43124, Italy; d Chemical Technologies Laboratory, DPIA, University of Udine, Udine 33100, Italy; e Department of Industrial Chemistry, University of Bologna, Bologna 40129, Italy

## Abstract

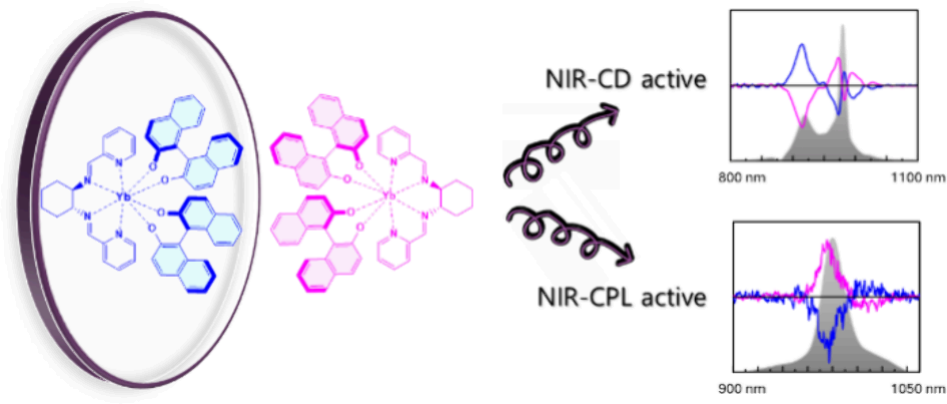

In this contribution, we present new NIR-CPL/CD active Yb(III) complexes, which are synthesized by combining two ligands characterized by different types of chirality: central and axial chirality. The ligand bearing central chirality is represented by the neutral *trans*-*N*,*N*′-bis(2-pyridylmethylidene)-1,2-(*R*,*R* or *S*,*S*) cyclohexanediamine (**L**), whereas the axial ligand is 1,1′-binaphthyl-2,2′-diol (BINOL-H_2_). A combined ^1^H NMR, thermodynamic (spectrophotometric titrations), and theoretical (DFT structural calculations) study is presented, revealing the high stability in the methanol solution of the homochiral *C*_*2*_-symmetric anionic [Yb**L**(BINOL)_2_]^−^ complexes, in which the two labile BINOLate molecules exhibit the same stereochemistry (*R* or *S*). The metal ion-related optical and chiroptical properties of the complexes in the near-infrared spectral region are here presented and discussed: while the axial chirality dominates the circularly polarized luminescence features, the NIR-CD signal is affected by both chiral elements.

## Abstract 2

[Yb**L**(BINOL)_2_]Na complexes are characterized by the axial chiral BINOLate ligands and by the central chiral **L** one. The two typologies of chirality differently affect the NIR-CPL and NIR-CD activity of Yb(III) ion.

## Introduction

It is well-known that the 650–1400 nm range, called the biological window, is a very attractive region for biological imaging.^1−7^ The emission of near-infrared (NIR) emitting Ln(III) complexes^8−13^ falls in this spectral region where common biological tissues are transparent and the radiations cause reduced damage due to photobleaching.^14^ Circularly polarized luminescence (CPL) in this region should benefit from the specificity, selectivity, and sensitivity, which are related to the chiroptical counterpart of emission spectroscopy.^3,15−17^

Moreover, NIR-CPL^18,19^ could expand the potential applications of these complexes to the technological field, making Ln(III) species good candidates also for optoelectronic devices,^20,21^ OLED,^22,23^ or laser systems.^24^

An efficient NIR-CPL activity is expected in the case of chiral Yb(III)-based complexes in which the spin allowed ^2^F_5/2_ → ^2^F_7/2_ emission transition, around 980 nm, is characterized by a high value of the rotational strength and sizable value of the emission dissymmetry factor *g*_Em_.^25^ In addition, in order to increase the emission intensity of Ln(III) ions, suitable organic sensitizers are usually linked to the metal center, triggering the well-known nonradiative ligand-to-metal energy transfer processes (*antenna* effect).^26−28^

**Figure 1:**
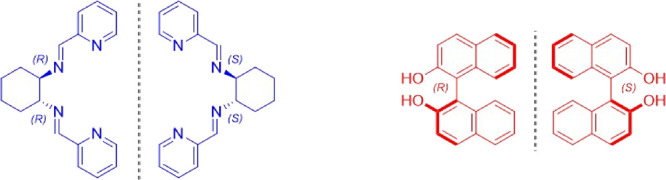
Central chiral ligand **L** (left) and axial chiral ligand BINOL-H_2_ (right).

One common drawback affecting the luminescence emission efficiency of NIR emitting Ln(III) complexes is represented by multiphonon relaxation (MPR) processes involving the stretching vibrations of the X-H bonds (X = C, N, O) present in the coordinating ligands and solvent molecules.^29^ Therefore, in an aqueous solution, water molecules in the proximity of the metal ion can significantly reduce the ^2^F_5/2_ excited state lifetime of Yb(III) and also its luminescence efficiency. The exclusion of water from the inner coordination sphere is strongly recommended to ensure a sufficient luminescence efficiency of the emitting metal ion. This has been achieved in cases such as a bicapped Yb(III) ion, utilizing a porphyrin-based double-decker^30^ or with hydrophobic complexes.^31^ Moreover, the detrimental effect of MPR can be significantly limited by embedding luminescent complexes in polymeric nanoparticles. Previous studies have demonstrated that poly lactic-*co*-glycolic acid (PLGA) is an attractive candidate for encapsulating and thus protecting Yb(III) complexes.^32^ Several years ago, Arrico et al. proposed to quantify the overall efficiency of circularly polarized emitters by means of the circularly polarized luminescence brightness (*B*_CPL_) factor. This efficiency is dependent not only on the degree of the CP emission’s dissymmetry (the relative amount of emitted left- compared to right-handed photons) but also on the molar extinction coefficient and the quantum yield of the compound.^33^ In other words, during the design of an efficient CPL emitter based on Ln(III) complexes, strong absorption of the chromophore, an efficient *antenna* effect, and negligible nonradiative phenomena like MPR must be considered. As for the ligands conferring CPL activity to Yb(III) ion, we focus our attention on two kinds of organic molecules: the neutral *trans*-*N*,*N*′-bis(2-pyridylmethylidene)-1,2-(*R*,*R* or *S*,*S*) cyclohexanediamine (**L**) bearing two stereocenters and the well-known 1,1′-binaphthyl-2,2′-diol (BINOL-H_2_)^34−37^ exhibiting axial chirality (Figure 1).

Yb(III) ion displays a *g*_lum_ factor (2(*I*_L_ – *I*_R_)/(*I*_L_ + *I*_R_)) of |0.03| and |0.17| when the first or the second ligand is employed in [Yb**L**(tta)_2_]CH_3_COO; tta = 2-thenoyltrifluoroacetonate^32^ and [(Binol)_3_YbNa_3_], respectively.^38^

The idea at the basis of the present study is to combine the two different kinds of stereogenic elements (central and axial chirality) to modulate the lanthanide-centered chiroptical features (NIR-CD and CPL, in particular).

To the best of our knowledge, this combination is performed for the first time in the literature in the case of Yb(III) complexes.

**Figure 2:**
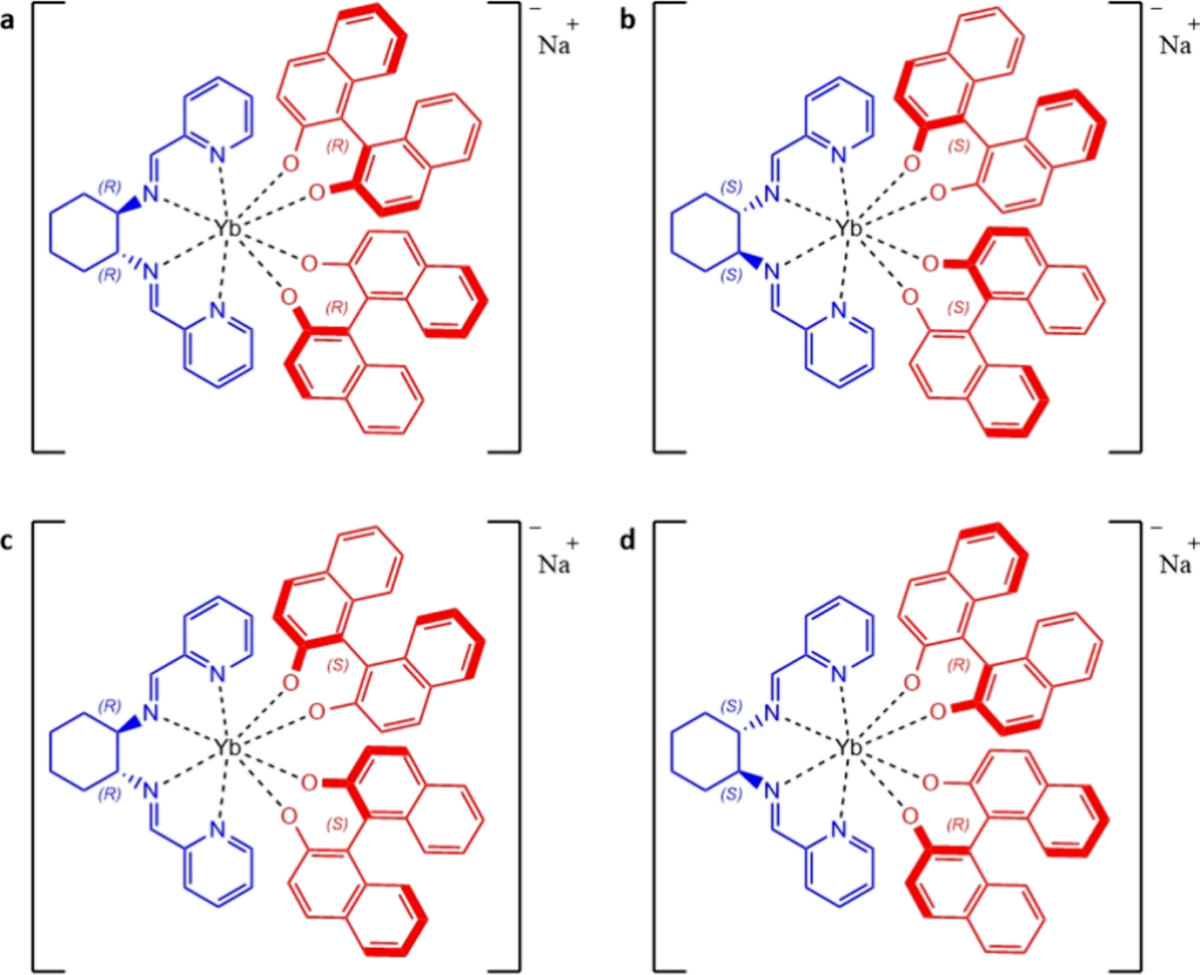
Complexes investigated in this work: (a) [Yb(*R,R*)-**L**(*R*-BINOL)_2_]Na, (b) [Yb(*S,S*)-**L**(*S*-BINOL)_2_]Na, (c) [Yb(*R,R*)-**L**(*S*-BINOL)_2_]Na, and (d) [Yb(*S,S*)-**L**(*R*-BINOL)_2_]Na.

In detail, by binding these two ligands (one equivalent of **L** and two equivalents of the deprotonated form BINOL^2–^) to the Yb(III) ion, several stereochemical combinations are in principle possible, all producing negatively charged complexes. In particular, focusing attention on BINOL stereochemistry, two diastereoisomeric homochiral species (i.e., [Yb(*R*,*R*)-**L**(*S*-BINOL)_2_]^−^ and [Yb(*R*,*R*)-**L**(*R*-BINOL)_2_]^−^ and their enantiomers) and one heterochiral enantiomers pair (i.e., [Yb(*R*,*R*)-**L**(*S*-BINOL)(*R*-BINOL)]^−^ and [Yb(*S*,*S*)-**L**(*R*-BINOL)(*S*-BINOL)]^−^ could be obtained. In this contribution, we demonstrate the higher stability of the homochiral species compared with the heterochiral species (Figure 2).

The stability of these complexes in methanol solution was determined as well as their DFT minimum energy structure. A detailed optical and chiroptical characterization in the NIR spectral region at around 1000 nm is presented.

## Experimental Section

Solvents were purchased from Fisher Scientific, VWR Chemicals, or Carlo Erba Reagents and used without further purification. Starting materials were purchased from Sigma-Aldrich and Alfa Aesar. Yb(NO_3_)_3_·5H_2_O (Aldrich, 98%) has been stored under vacuum for several days at 80 °C and then transferred into a glovebox.

### Elemental Analysis

Total carbon, hydrogen, nitrogen, and sulfur concentrations were determined by flash combustion using an elemental analyzer (CHNS vario Macro Cube, Elementar, Germany). Sulfanilamide (Elementar Analysensysteme GmbH, Germany) was used as a standard. The QA/QC was generally >96% for all elements.

### Infrared Absorption

IR spectra were collected by means of a PerkinElmer FT-IR spectrum two spectrophotometer (resolution of 4 cm^–1^).

### ESI-MS

Electrospray ionization mass spectra (ESI-MS) were recorded in methanol solutions on a Waters Micromass ZQ 4000 instrument operating in positive ion mode. Experimental conditions: 3.53 kV ES-probe voltage, 20 V cone potential, 200 L h^–1^ flow of N_2_ spray-gas, and incoming-solution flow of 20 μL min^–1^.

### NMR

^1^H NMR spectra were recorded on a Varian Mercury 400 or Inova 600 spectrometer. Chemical shifts (*d*) are reported in parts per million relative to TMS. Coupling constants (*J*) are reported in Hertz (Hz). Splitting patterns are designated as s (singlet), d (doublet), t (triplet), q (quartet), and m (multiplet).

### Total Luminescence (TL)

The emission and decay time measurements were carried out by means of an Edinburgh FLS1000 spectrofluorometer equipped with both continuous and pulsed Xe lamps, a double excitation monochromator, a single emission monochromator, and a N_2_-cooled NIR extended photomultiplier for the detection of the emitted signal. All the spectra were measured at room temperature and corrected for the spectral responsiveness of the setup.

### NIR-CPL Measurements

NIR-CPL spectra were recorded using the home-built spectrofluoropolarimeter, equipped with a Hamamatsu, R316 Ag–O–Cs photomultiplier tube, as described in ref (20). The spectra were collected under 365 nm irradiation from a commercial LED source, using a 90° geometry between the excitation and detection direction. All the NIR-CPL spectra were recorded on 1 mM CD_3_OD solutions in 1 cm semimicro (aperture 4 mm) optical glass cells using the following parameters: scan speed of 0.5 nm/s, integration time of 4 s, photomultiplier tube driving voltage of 1100 V, accumulations of 6–9, and em. bandwidth of >10 nm.

### UV–vis/ECD (Electronic Circular Dichroism) and NIR-CD Measurements

UV–vis spectra were recorded using a Jasco-V650 spectrophotometer in the spectral range of 200 to 400 nm. All samples were measured in 1 mM CD_3_OD solutions at room temperature in a 0.01 cm optical glass cell. The same solutions and cells were used to record CD spectra using a J1500 spectropolarimeter. NIR-CD measurements were performed by using a Jasco J-1500 spectropolarimeter equipped with an NIR detector (EXIG-542). Complexes were measured in DCM of 10 mM due to improved solubility. All complexes were measured in 1 cm optical glass cells with parameters as follows: scan speed of 100 nm/min, bandwidth of 10 nm, integration time of 4 s, and accumulations of 8.

### DFT Calculations

All molecular structures of the complexes were obtained by means of DFT calculations run in Gaussian 16 (version A.03).^39^ The ωB97X–D functional^40^ was used with the 6-31+G(d) basis set for all ligand atoms and MWB28 pseudopotential and valence electrons basis set for the metal ion.^41^ The paramagnetic Yb(III) ion was replaced by a similar Lu(III) ion. As for structural properties and the differences in the total energies, the Lu(III) ion can be considered the best computational model for Yb(III) since it has almost the same ionic radius as Yb(III), but its closed shell electron configuration enables to run less computationally demanding calculations. Geometry optimizations were carried including solvent (methanol) effects by means of the polarizable continuum model (PCM).^42^

Vibrational analysis was performed to confirm that the obtained structures were energy minima.

### Synthesis of the Complexes

The *trans*-*N*,*N*′-bis(2-pyridylmethylidene)-1,2-(*R*,*R* or *S*,*S*) cyclohexanediamine ligand (**L**) was synthesized as reported in the literature.^43^

[Yb(**L**)(BINOL)_2_]Na isomers (Figure 2) were synthesized as follows. At room temperature, Yb(NO_3_)_3_·5H_2_O (80 mg, 0.18 mmol) was added to a clear light brown methanol solution of *trans*-*N*,*N*′-bis(2-pyridylmethylidene)-1,2-(*R,R* or *S,S*) cyclohexanediamine (**L**) (52 mg, 0.18 mmol). In another flask, 1,1′-binaphthyl-2,2′-diol (BINOL-H_2_) (*S* or *R*) (102 mg, 0.36 mmol) was solubilized in methanol and added to a solution of NaOMe (39 mg, 0.72 mmol) in the same solvent. The deprotonated BINOL ligand was slowly added to the cloudy light brown solution containing the Ln(III) complex. The final yellow and cloudy mixture was stirred for 3 h. Then, the solvent was removed under reduced pressure, and the desired product was obtained as a yellow powder upon extraction in dichloromethane (2 × 7 mL) followed by solvent removal in vacuo. The chemical yield was around 90%, for the isomers mixture, which was obtained with a high degree of purity, as demonstrated by the following characterization analysis.

The Y(III) counterpart of the isomer c (Figure 2) [Y(*R,R*)-**L**(*S*-BINOL)_2_]Na was synthesized by following the aforementioned protocol but using YCl_3_ instead of Yb(NO_3_)_3_. The chemical yield was 85%.

Elemental analysis:calc. for C_58_H_46_N_4_NaO_5_Yb (MW 1075.07 g/mol), corresponding to [Yb(**L**)(BINOL)_2_]Na·H_2_O: C, 64.80; H, 4.31; N, 5.21. Found (isomer a): C, 64.91; H, 4.27; N, 5.24; (isomer b): C, 64.85; H, 4.28; N, 5.26; (isomer c): C, 64.76; H, 4.25; N, 5.25; (isomer d): C, 64.75; H, 4.29; N, 5.26. Calc. for C_58_H_46_N_4_NaO_5_Y (MW 990.92 g/mol), corresponding to [Y(**L**)(BINOL)_2_]Na·H_2_O: C, 70.30; H, 4.68; N, 5.65. Found: C, 70.52; H, 4.81; N, 5.58.

In the infrared-absorption spectrum of [Yb(*S*,*S*)-**L**(*R*-BINOL)_2_]Na (chosen as representative) (Figure S1) the signals related to OH stretching of *R*-BINOL-H_2_ at ν_OH_ = 3500 and 3420 cm^–1^ disappear. The signal related to the C=N stretching (ν_C=N_ = 1650 cm^–1^) is present corroborating the presence of the imine functional group of the **L** ligand in the complex.

ESI-MS (scan ES+, *m*/*z*) [Yb**L**(BINOL)_2_][Na]: 1058 {[Yb**L**(BINOL)_2_][H][Na]}^+^, 1036 {[Yb**L**(BINOL)_2_][H]_2_}^+^, 782 {[Yb(BINOL)_2_][H][K]}^+^, 750 {Yb**L**(BINOL)}^+^ for all the isomers (see Figure S2 and Table S1). [Y**L**(BINOL)_2_][Na]: 973 {[Y**L**(BINOL)_2_][H][Na]}^+^, 951 {[Y**L**(BINOL)_2_][H]_2_}^+^, 697 {Y**L**(BINOL)CH_3_OH}^+^, 665 {Yb**L**(BINOL)}^+^ (Figure S3 and Table S2).

## Results and Discussion

### Density Functional Theory (DFT) Calculation and ^1^H NMR Investigation

**Figure 3:**
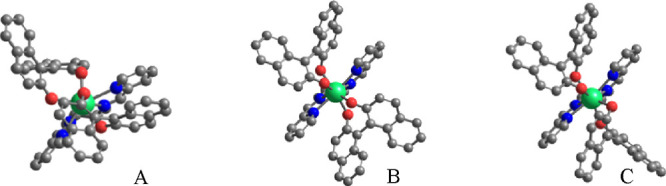
Minimum energy structures of (A) [Lu(*R*,*R*)-**L**(*R*-BINOL)_2_]^−^, (B) [Lu(*R*,*R*)-**L**(*S*-BINOL)_2_]^−^, and (C) [Lu(*R*,*R*)-**L**(*R*-BINOL)(*S*-BINOL)]^−^. Additional images with different orientations can be found in Figure S7.

In the absence of single crystals of good quality for the crystal structure determination by means of X-ray diffraction, density functional theory (DFT) calculations were performed on three representative species, whose minimum energy structures are reported in Figure 3.

In all cases, the metal ion (Lu was used to model Yb) displays an 8-fold coordination characterized by the four nitrogen atoms of the DACH-based **L** ligand and four oxygen atoms (two atoms for each BINOL molecule). The two BINOL ligands are bound above and below the plane defined by the **L** ligand.

Regarding the configuration of the binaphthol units, the calculated difference in energy between the homochiral [Lu(*R*,*R*)-**L**(*R*-BINOL)_2_]^−^ (A) and [Lu(*R*,*R*)-**L**(*S*-BINOL)_2_]^−^ (B) species (Figure 3) is Δ*E* = *E*(B) – *E*(A) = 1.3 kcal mol^–1^ suggesting similar stability of the two isomers. On the contrary, the heterochiral [Lu(*R*,*R*)-**L**(*R*-BINOL)(*S*-BINOL)]^−^ species (C) is significantly less stable [Δ*E* = *E*(C) – *E*(A) = 3.2 kcal mol^–1^] and its presence in a complex solution containing Yb^3+^, (*R*,*R*)-**L**, (*R*-BINOL), and (*S*-BINOL) can be in principle neglected.

In order to gain more insight into the structure of the Yb(III) complexes in solution, we performed a ^1^H NMR analysis. Since the resulting spectra were characterized by very broad peaks and therefore they were not informative, we investigated the ^1^H NMR spectra of the representative Y(III) complexes, i.e., [Y(*R*,*R*)-**L**(*S*-BINOL)_2_]Na. The diamagnetic properties of Y(III) will help to obtain more information about the nature and the structure of the complex, considering the very small difference of the ionic radii of Yb(III) and Y(III) (0.03 Å for 8-fold coordination), which makes the Y(III) a suitable structural equivalent of Yb(III). The [Y(*R*,*R*)-**L**(*S*-BINOL)_2_]Na complex was analyzed using ^1^H NMR (Figure S4) and gCOSY ^1^H-{^1^H} (CD_2_Cl_2_ solution) (Figure S5). As detailed in the Supporting Information (Section 1.3), while the pyridinic, iminic, and methinic protons are readily identifiable in the spectrum and suggest a *C*_2_ symmetry of the molecule, some signals related to aromatic protons of the BINOLate ligands are very broad, and only five aromatic protons (H_Ar_) are clearly identified in the spectrum (Figure S4). The broadening of these signals should be strongly connected with the lability of metal-BINOL bonds in solution as previously observed for the Yb(III) ion.^44,45^ In order to resolve the broad aromatic peaks of BINOLate, variable temperature (VT) ^1^H NMR experiments in CD_3_CN have been carried out in the range from −30 to 65 °C as shown in Figure S6. Despite this attempt, only a partial resolution of the two broad aromatic bands has been obtained upon heating the sample, so the integration of these signals cannot be used to confirm the stoichiometry of the complex, which has been determined by means of other experimental techniques (elemental analysis and ESI-MS).

### Thermodynamic Study

To experimentally determine the stability of the homochiral complexes, spectrophotometric titrations in the methanol solution of both BINOL enantiomers with [Yb(*R*,*R*)-**L**](NO_3_)_3_ (Yb(*R*,*R*)-**L** in Table 1) were carried out (Figure S9). As suggested by DFT calculations, the two homochiral complexes, [Yb(*R*,*R*)-**L**(*R*-BINOL)_2_]^−^ and [Yb(*R*,*R*)-**L**(*S*-BINOL)_2_]^−^, show a very similar stability (Table 1).

**Table 1:** Formation Constants (logβ) in Dry Methanol for the Yb(III) Complexes under Investigation

**equilibria**	**logβ**
Yb(*R*,*R*)-**L** + *S*-BINOL ⇋ Yb(*R*,*R*)-**L**(*S*-BINOL)	7.57 (±0.07)
Yb(*R*,*R*)-**L** + 2*S*-BINOL ⇋ Yb(*R*,*R*)-**L**(*S*-BINOL)_2_	13.3 (±0.1)
Yb(*R*,*R*)-**L** + *R*-BINOL ⇋ Yb(*R*,*R*)-**L**(*R*-BINOL)	7.87 (±0.04)
Yb(*R*,*R*)-**L** + 2*R*-BINOL ⇋ Yb(*R*,*R*)-**L**(*R*-BINOL)_2_	13.2 (±0.1)

### Total Luminescence (TL) and Luminescence Decay Kinetics

**Figure 4:**
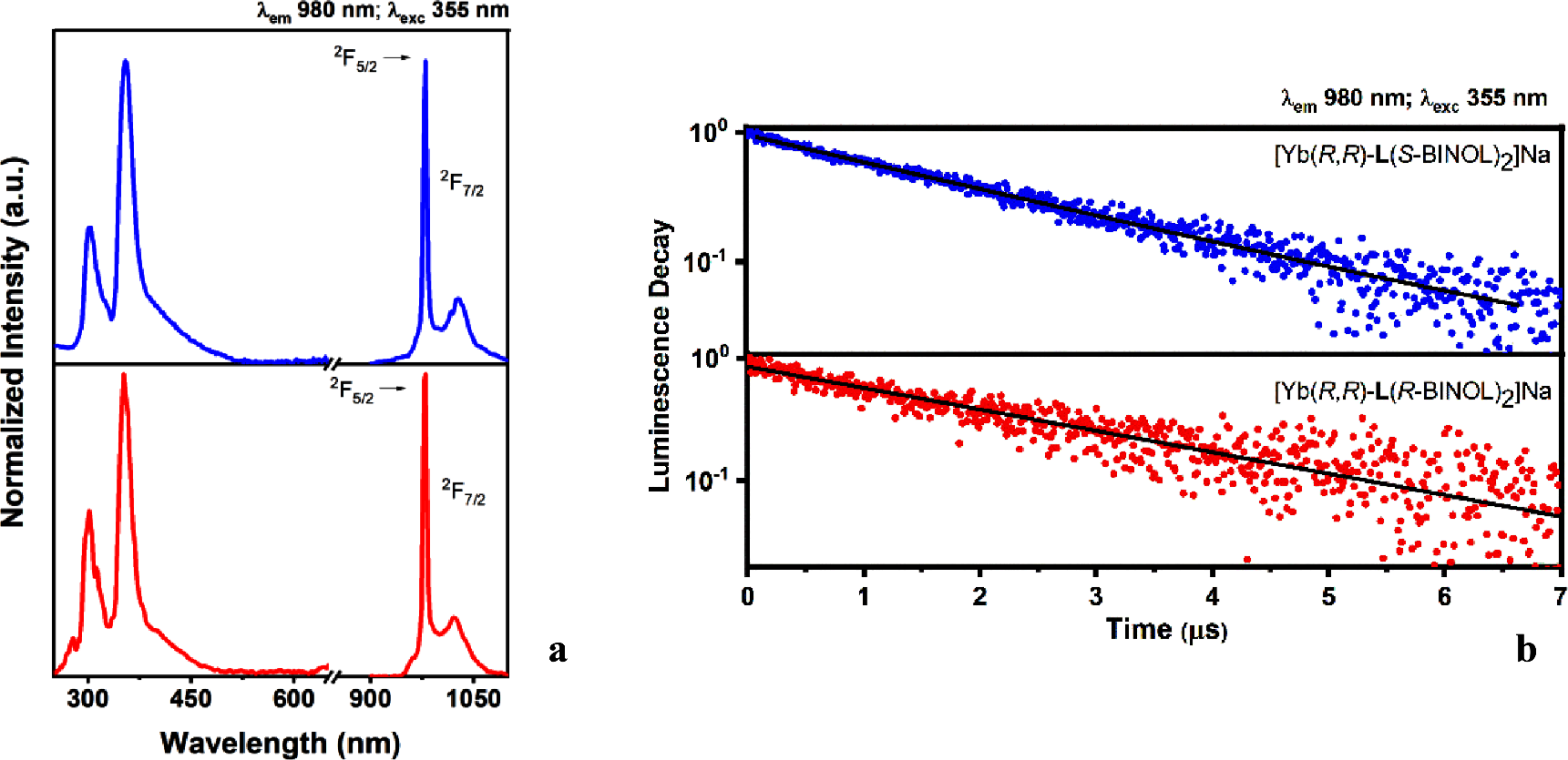
(a) Excitation (left) and emission (right) spectra in methanol solution (200 μM) of the two homochiral complexes [Yb(*R*,*R*)-**L**(*S*-BINOL)_2_]Na (up) and [Yb(*R*,*R*)-**L**(*R*-BINOL)_2_]Na (bottom). These spectra are fully superimposable with those of the respective enantiomers [Yb(*S*,*S*)-**L**(*R*-BINOL)_2_]Na and [Yb(*S*,*S*)-**L**(*S*-BINOL)_2_]Na; (b) luminescence decay curves of the Yb(III) ^2^F_5/2_ excited state for methanol solutions (200 μM) of [Yb(*R*,*R*)-**L**(*S*-BINOL)_2_]Na and [Yb(*R*,*R*)-**L**(*R*-BINOL)_2_]Na complexes under investigation, chosen as representative. These curves are fully superimposable with those of the respective enantiomers [Yb(*S*,*S*)-**L**(*R*-BINOL)_2_]Na and [Yb(*S*,*S*)-**L**(*S*-BINOL)_2_]Na.

From the inspection of the absorption spectra of the complexes (Figure S10), the band just below 300 nm (ε_max_ = 27000 M^–1^cm^–1^) may be attributed to transitions of both BINOL^–2^ and pyridine ring of **L** (the π → π* transition),^32^ whereas the more red-shifted band close to 350 nm (ε_max_ = 12000 M^–1^cm^–1^), to one of the π → π* of the BINOL^–2^.^44^ Furthermore, the luminescence excitation and emission spectra of methanol solutions (200 μM) of the complexes are shown in Figure 4a.

The excitation profiles demonstrate the presence of an efficient *antenna* effect of both ligands, in particular for BINOL. In fact, the intensity of the peak located around 350 nm is almost twice that of the peak at 300 nm.

The typical luminescence emission spectra of Yb(III) are obtained,^32^ dominated by a sharp transition peaking at 980 nm and a more complex manifold in the 900–1100 nm region, associated to the crystal field splitting of the ^2^F_7/2_ ground state. Upon excitation in the BINOL absorption band (355 nm), the recorded luminescence decay of the ^2^F_5/2_ excited state of Yb(III) (monitored at 980 nm) in the two samples is similar and follows an exponential decay (Figure 4b). The related lifetimes are 2.49(4) and 2.06(3) μs for [Yb(*R*,*R*)-**L**(*R*-BINOL)_2_]Na and [Yb(*R*,*R*)-**L**(*S*-BINOL)_2_]Na, respectively.

As reported in the literature,^46−48^ the values of these decay times are compatible with the presence of solvent molecules (i.e., polar protic solvents) in the proximity of Yb(III) capable of quenching its excited state *via* MPR.^49,50^ In particular, as the OH group of methanol has high-energy vibrations (3300–3400 cm^–1^), it can efficiently quench the Ln(III) emitting level.^51^

The slightly shorter lifetime of the [Yb(*R*,*R*)-**L**(*S*-BINOL)_2_]Na complex can be rationalized by the shorter distance between the solvent molecules and the metal center, found by DFT calculations on the related Lu(III) complexes in the presence of solvent molecules (Figure S8, water was used as a model for methanol). In detail, while in [Lu(*R*,*R*)-**L**(*R*-BINOL)_2_]Na one solvent molecule lies in the outer coordination sphere, in [Lu(*R*,*R*)-**L**(*S*-BINOL)_2_]Na, the solvent molecule is closer to the inner coordination sphere, as the solvent is bound, through hydrogen bond, to one oxygen atom of BINOL. In this context, the closer the solvent molecule is to the metal ion, the more efficient the MPR process is and therefore shorter the Yb(III) lifetime.

### Chiroptical Spectroscopy

**Figure 5:**
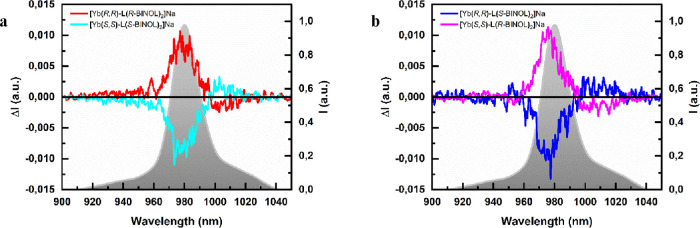
NIR-CPL spectra of 1 mM CD_3_OD solutions of the two enantiomeric couples of the investigated Yb(III) complexes. (a) [Yb(*R*,*R*)-**L**(*R*-BINOL)_2_]Na (red)/[Yb(*S*,*S*)-**L**(*S*-BINOL)_2_]Na (cyano) and (b) [Yb(*R*,*R*)-**L**(*S*-BINOL)_2_]Na (blue)/[Yb(*S*,*S*)-**L**(*R*-BINOL)_2_]Na (magenta). The normalized total emission for each couple is traced in the background. λ_exc_ = 365 nm.

The NIR-CPL spectra of chiral complexes [Yb(*R*,*R*)-**L**(*S*-BINOL)_2_]Na and [Yb(*R*,*R*)-**L**(*R*-BINOL)_2_]Na (and the related enantiomers) are reported in Figure 5.

**Figure 6:**
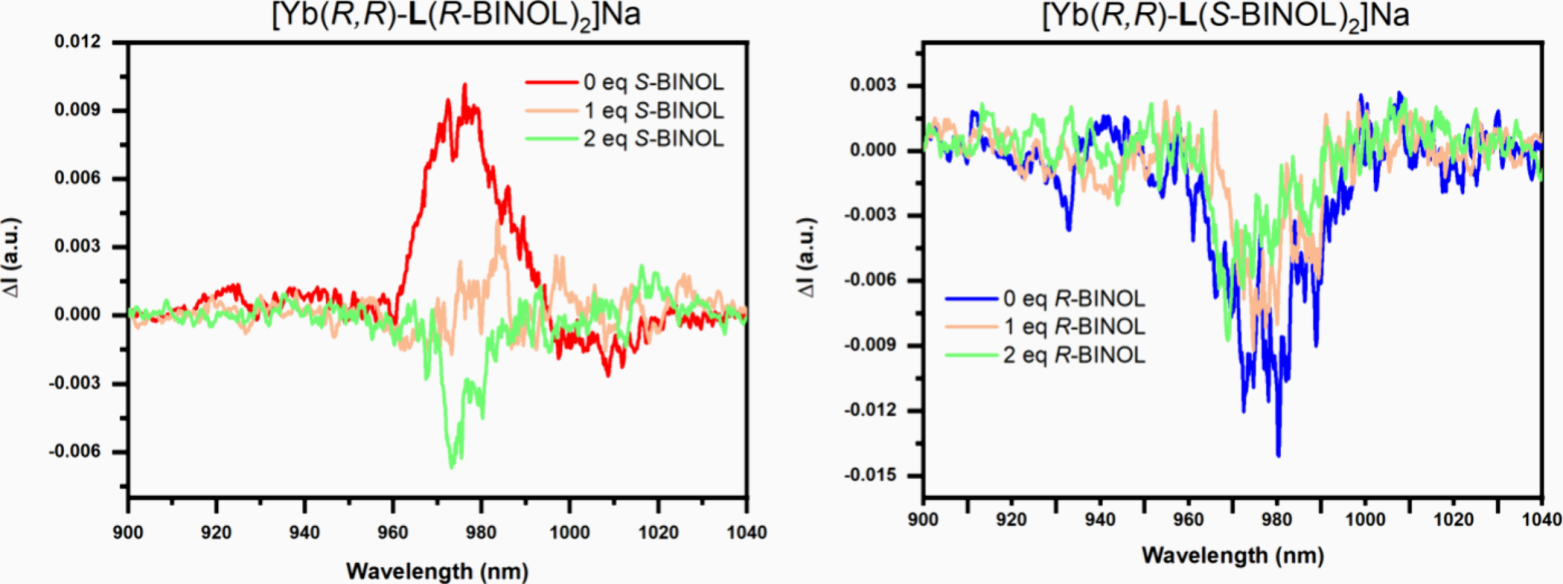
Changes of the CPL signature upon titration of [Yb(*R,R*)-**L**(*R*-BINOL)_2_]Na with *S*-BINOL (left) and of [Yb(*R,R*)-**L**(*S*-BINOL)_2_]Na with *R*-BINOL (right).

From the inspection of these spectra, we can conclude that the CPL activity of Yb(III) is mainly controlled by the BINOL stereochemistry. In fact, a negative band with a *g*_lum_ of 0.01 around 980 nm is detected when two *S*-BINOLs are bound to the metal center, regardless of the stereochemistry of the **L** ligand (please refer to the spectra of [Yb(*R*,*R*)-**L**(*S*-BINOL)_2_]Na and [Yb(*S*,*S*)-**L**(*S*-BINOL)_2_]Na in Figure 5). To corroborate the dominant role of the BINOL stereochemistry on the NIR-CPL activity of the Yb(III) ion, we performed CPL titration experiments in which the Yb(III) complexes were titrated with the BINOL enantiomer of opposite chirality with respect to the one present in the complex (i.e., [Yb(*R*,*R*)-**L**(*S*-BINOL)_2_]Na titrated with *R*-BINOL and [Yb(*R*,*R*)-**L**(*R*-BINOL)_2_]Na titrated with *S*-BINOL). In agreement with the lability of Yb-BINOL bonds in a solution of polar solvents previously discussed,^44,45^ a change in the CPL spectrum was observed (Figure 6). In both the titrations, the weak negative CPL band obtained after the addition of 2 equiv of BINOL is consistent with a slightly higher thermodynamic stability of [Yb(*R*,*R*)-**L**(*S*-BINOL)_2_]Na with respect to [Yb(*R*,*R*)-**L**(*R*-BINOL)_2_]Na (Table 1).

**Figure 7:**
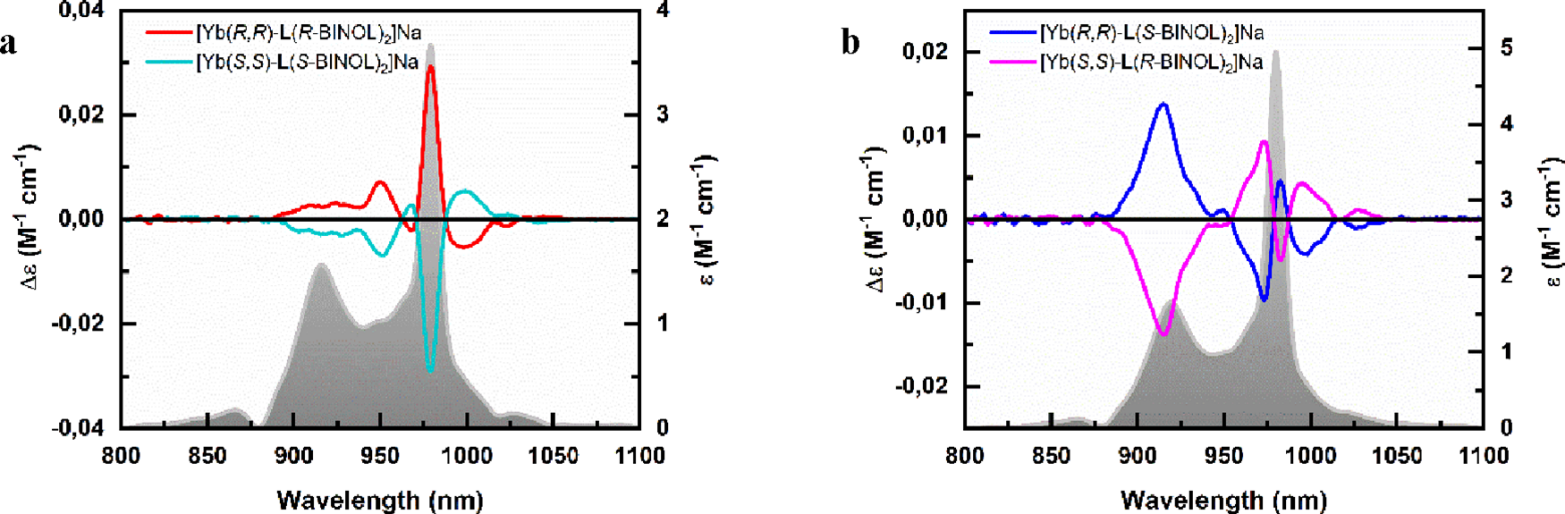
NIR-CD spectra of 10 mM CD_3_OD solutions of the two enantiomeric couples of the investigated Yb(III) complexes. (a) [Yb(*R*,*R*)-**L**(*R*-BINOL)_2_]Na (red)/[Yb(*S*,*S*)-**L**(*S*-BINOL)_2_]Na (cyano) and (b) [Yb(*R*,*R*)-**L**(*S*-BINOL)_2_]Na (blue)/[Yb(*S*,*S*)-**L**(*R*-BINOL)_2_]Na (magenta). The molar absorption coefficient as a function of the wavelength for each couple is traced in the background.

Contrary to the NIR-CPL activity, the NIR-CD and ECD in the UV region are significantly affected by both kinds of stereogenic elements (Figure 7; see also Figure S10 for ECD spectra).

When keeping the central stereochemistry of the ligand **L** constant, and upon switching the BINOL stereochemistry (passing from [Yb(*R*,*R*)-**L**(*R*-BINOL)_2_]Na to [Yb(*R*,*R*)-**L**(*S*-BINOL)_2_]Na), the relative intensity (but not the sign) of the CD peaks changes. On the other hand, the only change of the **L** stereochemistry (i.e., passing from [Yb(*R*,*R*)-**L**(*R*-BINOL)_2_]Na to [Yb(*S*,*S*)-**L**(*R*-BINOL)_2_]Na) gives rise to the reversal of the CD peaks signal while also affecting their relative intensities.

Moreover, it is interesting to note that NIR-CD data support what was deduced through DFT calculations about the instability of heterochiral [Yb(*R*,*R*)-**L**(*R*-BINOL)(*S*-BINOL)]^−^. More in detail, the NIR-CD signal of a 1:1:1:1 (molar ratio) solution containing Yb^3+^, (*R*,*R*)-**L**, (*R*-BINOL), and (*S*-BINOL) is superimposable to the sum of the NIR-CD signals of the two homochiral complexes (Figure S11), confirming that the existence of the heterochiral species can be neglected. We note that the relatively large bands observed in CPL are the result of a complex overlap of transitions, possibly stemming from different M_J_ levels above the 0’ of the excited ^2^F_5/2_ state, which may be populated at room temperature.^52^

## Conclusions

In conclusion, the synthesized *C*_*2*_-symmetric [Yb**L**(BINOL)_2_]Na complexes, characterized by the same stereochemistry of the two BINOL ligands (*S* or *R*), combine two kinds of chiralities (central and axial) in the same molecule. These stereogenic elements affect the chiroptical properties, such as CPL and CD of the Yb(III) ion: the Yb(III) CPL signal around 980 nm is mainly dictated by the BINOL stereochemistry (S → −; R → +), and the Yb(III) CD signal in the same spectral region is affected by the stereochemistry of both ligands over the entire region of the ^2^F_7/2_ → ^2^F_5/2_ transition. Additionally, CPL and ^1^H NMR techniques prove the lability of BINOLate ligands in these kinds of complexes. These very interesting features can be conveniently exploited to design an optical material with easily tunable chiroptical activity, in the so important (for biological and technological applications) NIR window. In fact, dramatic changes in CD and CPL spectra around 1 μm can be obtained by simply playing with the handedness of the two chiral ligands.

## Supporting Information Available

Characterization of the synthesized complexes: FT-IR of [Yb(*S,S*)-**L**(*R*-BINOL)_2_]Na (Figure S1), ESI-MS of [Yb(*R,R*)-**L**(*R*-BINOL)_2_]Na, [Yb(*R,R*)-**L**(*S*-BINOL)_2_]Na and the related enantiomers (Figure S2). ESI-MS of [Y(*R,R*)-**L**(*S*-BINOL)_2_]Na (Figure S3); ^1^H NMR (also VT), gCOSY spectrum ^1^H-{^1^H} NMR spectra of [Y(*R,R*)-**L**(*S*-BINOL)_2_]Na (Figures S4–S6); DFT calculation and thermodynamic study: minimum energy structures of [Lu(*R,R*)-**L**(*R*-BINOL)_2_]^−^, [Lu(*R,R*)-**L**(*S*-BINOL)_2_]^−^, and [Lu(*R,R*)-**L**(*R*-BINOL)(*S*-BINOL)]^−^ (Figures S7 and S8); spectrophotometric titrations of *S*-BINOL with [Yb(*R,R*)-**L**](NO_3_)_3_ and *R*-BINOL with [Yb(*R,R*)-**L**](NO_3_)_3_ (Figure S9); chiroptical spectroscopy: ECD spectra of Yb(*R,R*)-**L**(*R*-BINOL)_2_]Na and [Yb(*R,R*)-**L**(*S*-BINOL)_2_]Na and related enantiomers (Figure S10); experimental and simulated NIR CD spectra of Yb^3+^ complexes (Figure S11) (PDF)
