# Supplementary material for: Near Infrared–Circularly Polarized Luminescence/Circular Dichroism Active Yb(III) Complexes Bearing Both Central and Axial Chirality
Source: Inorg Chem. 2025 Mar 13;64(11):5505–12. doi: 10.1021/acs.inorgchem.4c05420 (PMC11938337; doi:10.1021/acs.inorgchem.4c05420)
Supplement: Supplementary file 1 — ic4c05420_si_001.pdf [file ic4c05420_si_001.pdf]

Supporting information

NIR-CPL/CD active Yb(III) complexes bearing both central and axial chirality

Silvia Ruggieri,[a*] Oliver George Willis,[b] Silvia Mizzoni,[a] Enrico Cavalli,[c] Martina Sanadar,[d] Andrea Melchior,[d] Francesco Zinna,[b] Lorenzo Di Bari,[b] Giorgiana Denisa Bisag,[e] Mariafrancesca Fochi,[e] Luca Bernardi,[e] and Fabio Piccinelli [a*]

[a] Luminescent Materials Laboratory, DB, University of Verona, and INSTM, UdR Verona, Strada Le Grazie 15, 37134 Verona, Italy
[b] Department of Chemistry and Industrial Chemistry, University of Pisa, via Moruzzi 13, 56124 Pisa, Italy
[c] Department of Chemistry, Life Sciences and Environmental Sustainability, University of Parma Parco Area delle Scienze, 17/a – 43124, Parma, Italy
[d] Polytechnic Department of Engineering, Laboratory of Chemical Technologies, University of Udine, via Cotonificio 108, 33100 Udine, Italy
[e] Department of Industrial Chemistry, University of Bologna, via Gobetti 85, 40129 Bologna, Italy

*corresponding authors' email address: silvia.ruggieri@univr.it; fabio.piccinelli@univr.it

LIST OF CONTENT

1. **Characterization of the synthesized complexes**
   1.1. Infrared spectra of Yb(III) species (Figure S1)
   1.2. ESI-MS spectra of Yb(III) species (Figure S2, Table S1) and of Y(III) species (Figure S3, Table S2)
   1.3. NMR spectra: ^{1}H NMR spectrum (Figure S4), gCOSY spectrum ^{1}H-{^{1}H} (Figure S5) and VT ^{1}H NMR spectra (Figure S6) of Y(III) species
2. **Density Functional Theory (DFT) calculation and Thermodynamic study**
   Minimum energy structures (Figure S7), Minimum energy structures with additional water molecule (Figure S8) of Lu(III) counterparts and Spectrophotometric titrations of Yb(III) species (Figure S9).
3. **Chiroptical spectroscopy**
   ECD spectra (Figure S10) and Overlap of experimental and simulated ECD spectra (Figure S11) of Yb(III) species

1. Characterization of the synthesized complexes

1.1. Infrared spectra

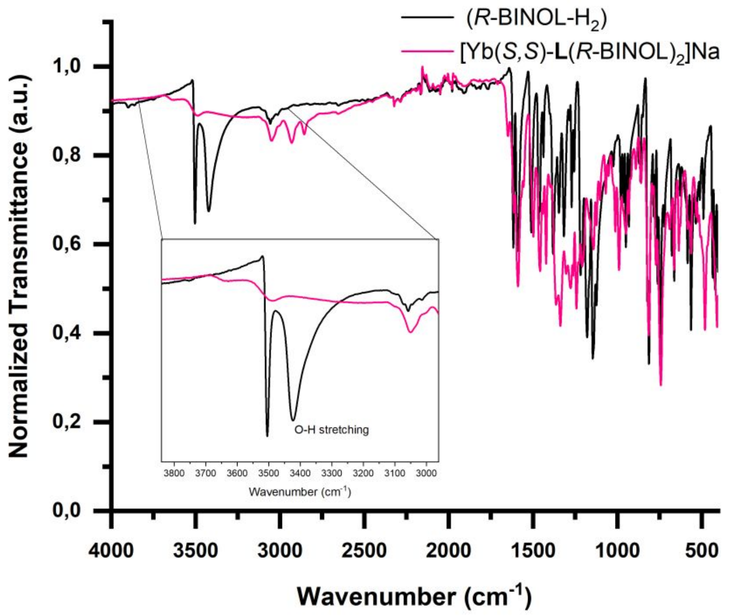

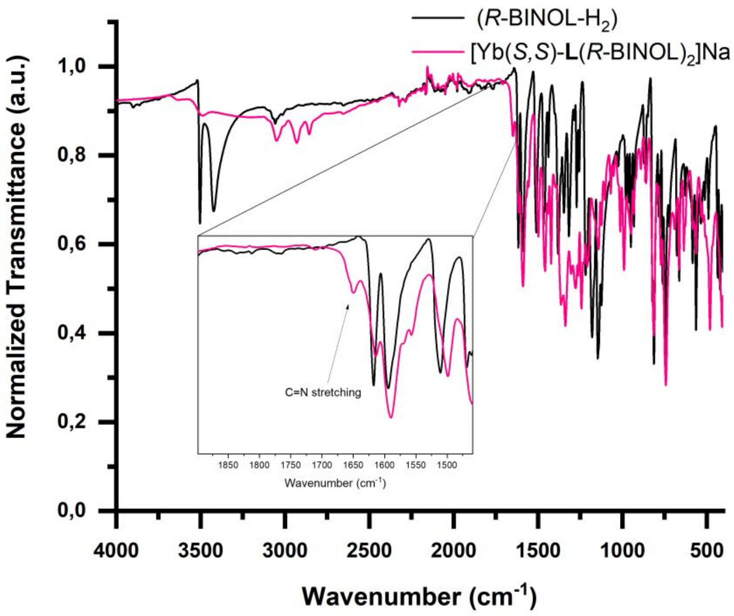

Figure S1. Normalized FT-Infrared absorption spectra of *R*-BINOL-H_2 and [Yb(*S,S*)-**L**(*R*-BINOL)$_2$]Na (chosen as representative), with (up) the OH stretching zoom (3420 cm^{-1}) of *R*-BINOL-H_2 and (bottom) the C=N stretching zoom (1650 cm^{-1}) due to the presence of the imine in **L** ligand.

1.2. ESI-MS spectra

[Yb(*R,R*)-**L**(*R*-BINOL)$_2$]Na

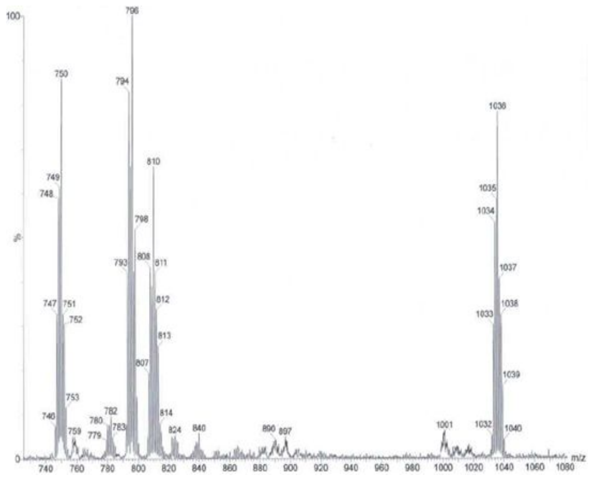

[Yb(*S,S*)-**L**(*S*-BINOL)$_2$]Na

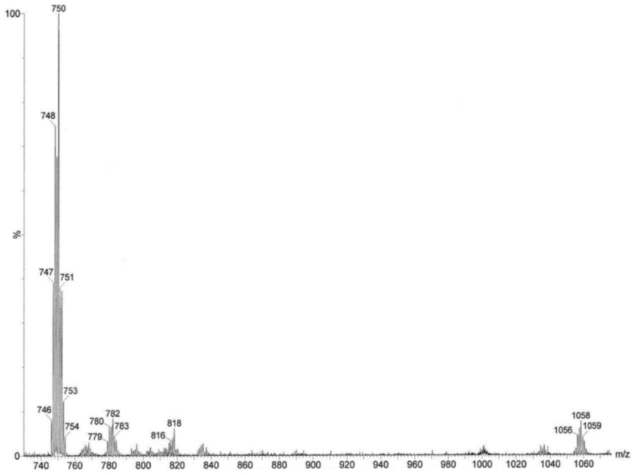

[Yb(*R,R*)-**L**(*S*-BINOL)$_2$]Na

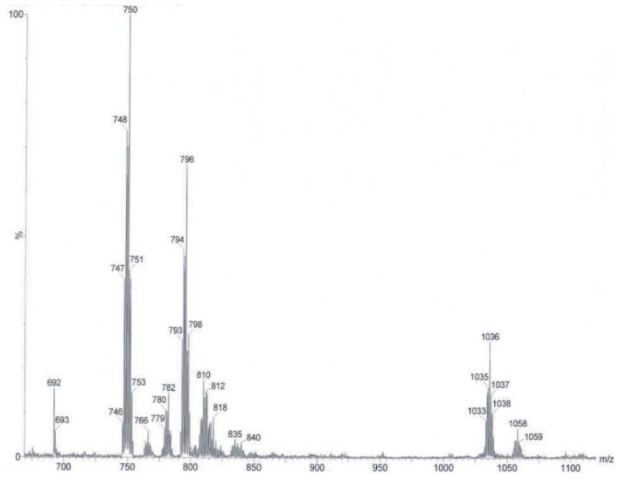

[Yb(*S,S*)-**L**(*R*-BINOL)$_2$]Na

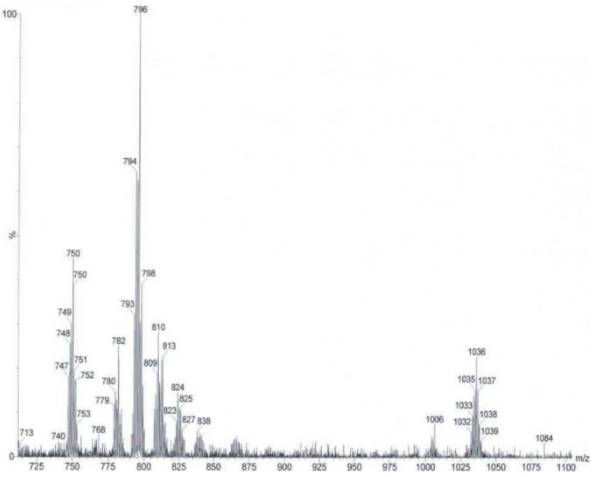

Figure S2.ESI-MS spectra of the synthesized homochiral Yb(III) complexes.

Table S1. ESI-MS peak assignments for the reported Yb(III) complexes.

Most relevant signals (m/z)	Corresponding Ions
1058	{[Yb**L**(BINOL)$_2$][H][Na]}$^+$
1036	{[Yb**L**(BINOL)$_2$][H]$_2$}$^+$
782	{[Yb(BINOL)$_2$][H][K]}$^+$
750	{Yb**L**(BINOL)}$^+$

[Y(*R,R*)-**L**(*S*-BINOL)$_2$]Na

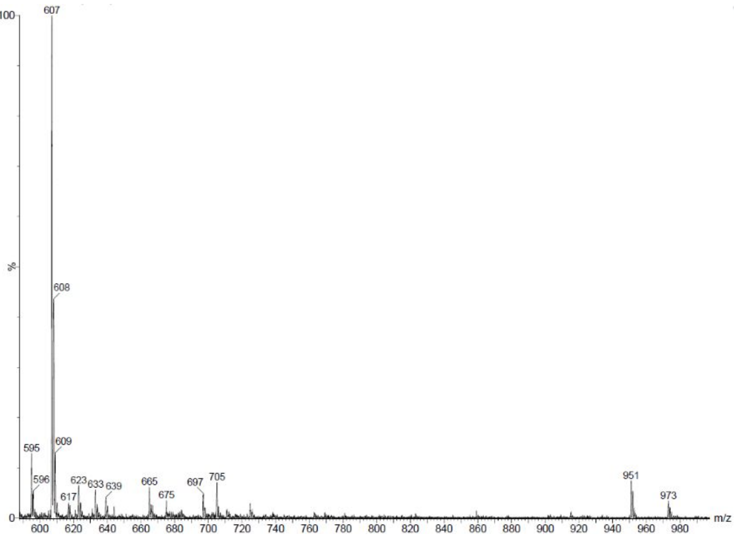

Figure S3.ESI-MS spectrum of the synthesized homochiral Y(III) complex.

Table S2. ESI-MS peak assignments for the reported Y(III) complex.

Most relevant signals (m/z)	Corresponding Ions
973	{[Y**L**(BINOL)$_2$][H][Na]}$^+$
951	{[Y**L**(BINOL)$_2$][H]$_2$}$^+$
697	{Y**L**(BINOL)CH_3OH}$^+$
665	{Y**L**(BINOL)}$^+$

1.3. NMR spectra

Yb(III) complexes cannot be analysed by NMR methods because most lanthanides contain a partially filled f-shell in their +3 oxidation state. The result is paramagnetism, which perturbs the magnetic properties of the nearby nuclei, rendering data acquisition and interpretation complicated at best, and often impractical. Yttrium, which shares similar chemical properties with the lanthanides, and is also classified as a “rare-earth-element”, offers a notable exception. In its most common +III oxidation state, yttrium is diamagnetic.

For this reason we synthetized the corresponding yttrium complex [Y(**L**)(*S*-BINOL)$_2$]Na following the same synthetic procedure starting from YCl_3. The resulting complex was analyzed using ^{1}H-NMR (Figure S4) and gCOSY ^{1}H-{^{1}H} (CD_2Cl_2 solution) (Figure S5).

As shown in Figure S4, the pyridinic protons are readily identifiable in the spectrum at 8.35 (br d, J = 8.3 Hz, 2H$_a$), 7,87 (br d, J = 7.9 Hz, 2H$_d$), 7.63 (td, J_1 = 7.8, J_2 = 1.8 Hz, 2H$_c$) and 7.17 (m, 2H$_b$), ppm. The iminic proton appears at 8.28 (br s, 2H$_e$) ppm while the cyclohexyl methinic proton is observed at 3.54 (m, 2H$_f$) ppm.

Regarding the BINOLate ligands, the aromatic protons (H$_{Ar}$) identified in the spectrum are located at 8.53 (br s, 2H), 7.79 (br t, 2H), 7.26 (br d, 2H), 7.16 (m, 3H) and 6.66 (br s, 2H). Moreover,

the spectrum shows two very broad signals in the aromatic region accounting for the other aromatic protons of the complex.

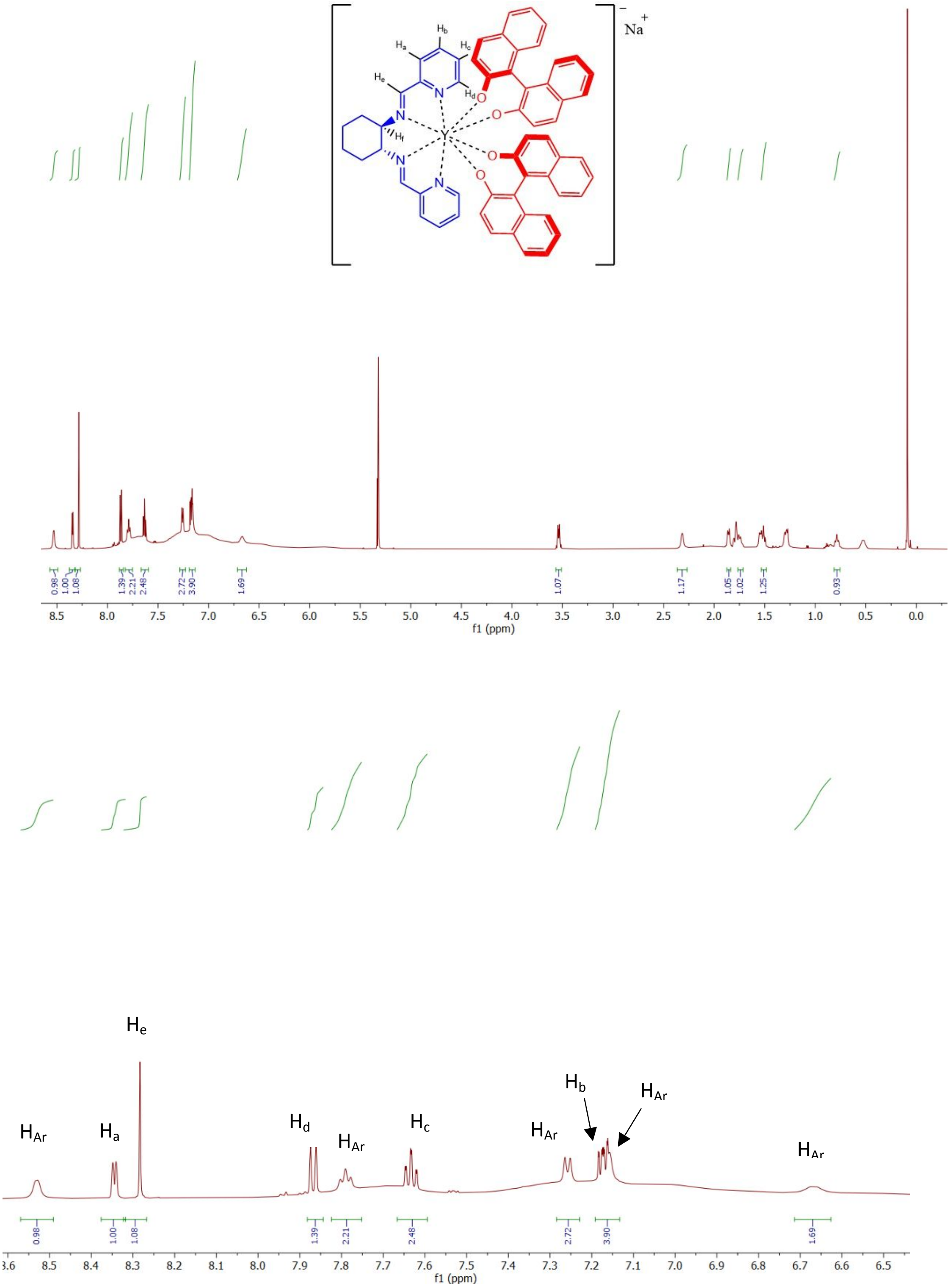

Figure S4.[Y(*R,R*)-**L**(*S*-BINOL)$_2$]Na 600MHz ^{1}H NMR spectrum in CD_2Cl_2 full spectrum (up); aromatic region (bottom).

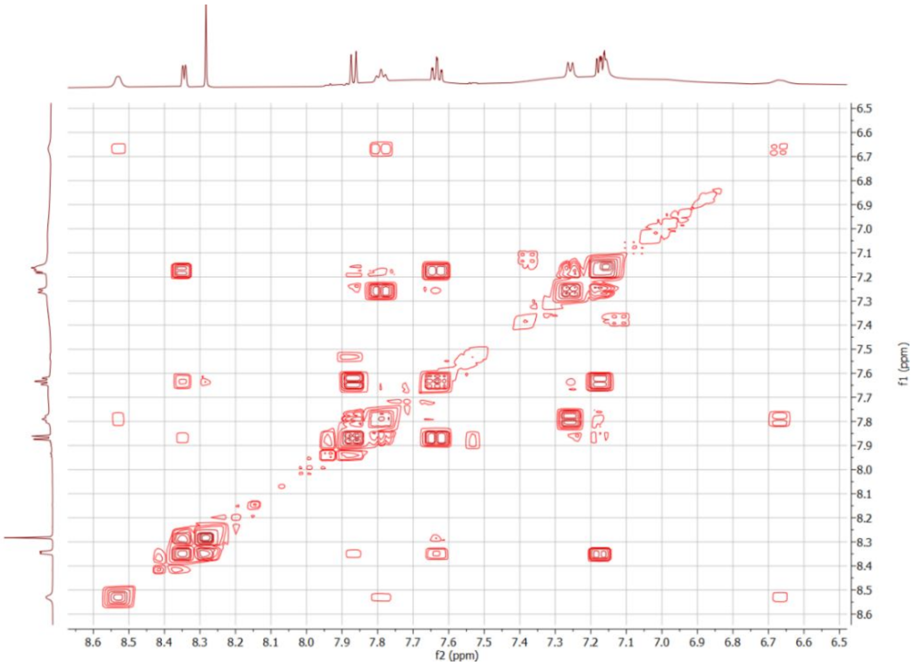

Figure S5. [Y(*R,R*)-**L**(*S*-BINOL)$_2$]Na 600MHz gCOSY spectrum ^{1}H-{^{1}H} in CD_2Cl_2 (aromatic region).

Variable temperature (VT) NMR experiments have been attempted to resolve broad aromatic peaks. ^{1}H NMR spectra in CD_3CN were recorded at temperatures ranging from - 30 to 65 °C as shown in Figure S6 and compared with the room temperature spectrum. Upon heating the sample, the two very broad aromatic peaks could be partially resolved, although some degree of overlap in the aromatic region persisted.

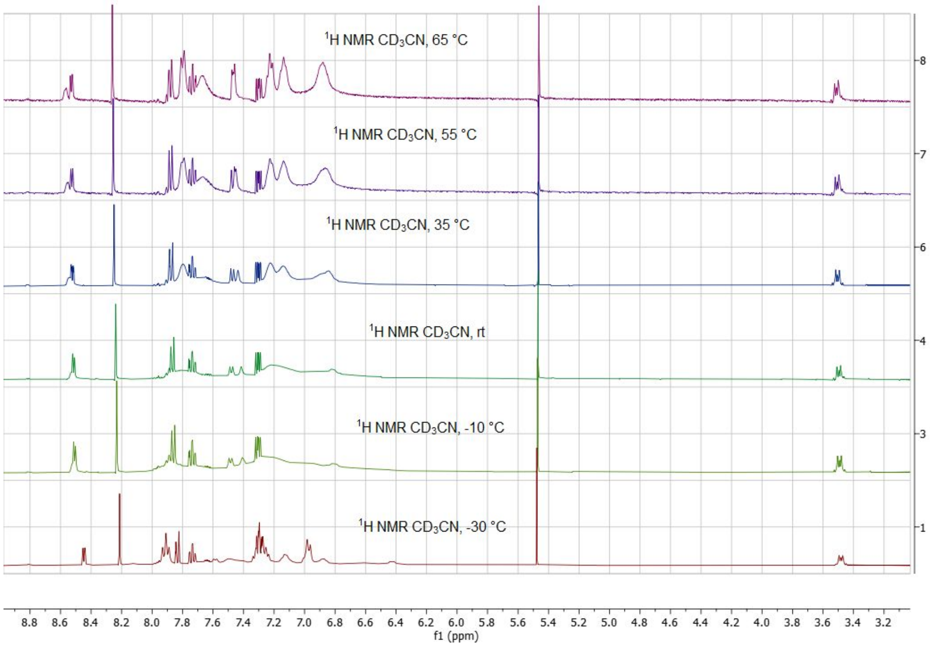

Figure S6. [Y(*R,R*)-**L**(*S*-BINOL)$_2$]Na 600MHz ^{1}H NMR spectra at different temperature in CD_3CN (aliphatic region 1-2.5 ppm have been omitted).

2. Density Functional Theory (DFT) calculation and Thermodynamic study

Isomer	E (kcal)	Front	Side	Rear
A	0.0	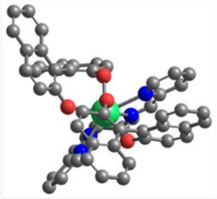	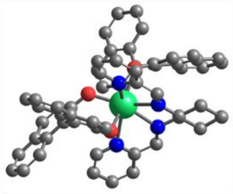	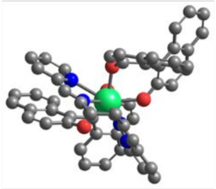
B	1.3	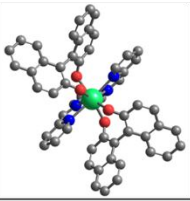	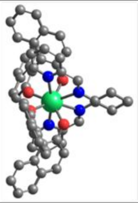	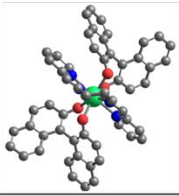
C	3.2	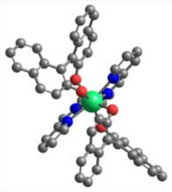	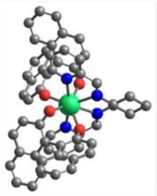	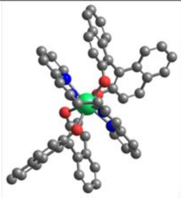

Figure S7. Minimum energy structures of the investigated complexes: A) [Lu(*R,R*)-**L**(*R*-BINOL)$_2$]$^-$ B) [Lu(*R,R*)-**L**(*S*-BINOL)$_2$]$^-$, C) [Lu(*R,R*)-**L**(*R*-BINOL)(*S*-BINOL)]$^-$. The relative energies (ΔE, kcal mol^{-1}) with respect to diastereoisomer A are reported. Hydrogen atoms were removed for clarity. The structures of the complexes are presented in three orientations keeping the central carbons of the DACH moiety in an equatorial plane as reference. Front: structure aligned with the DACH in the back with respect to the observer. Side: the structure is aligned along the DACH visible on the right. Rear: the structure is aligned along the DACH visible on the front.

A	B	C
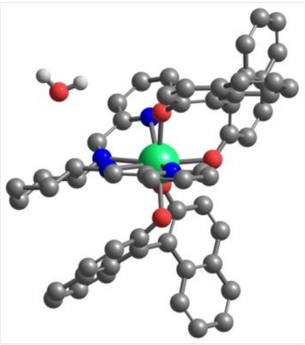	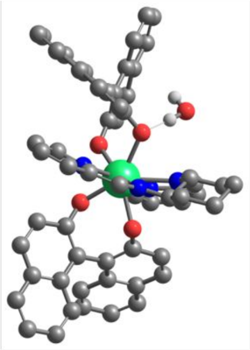	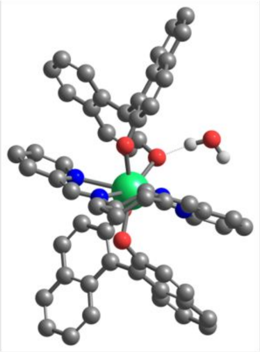
Lu-O$_{water}$= 4.24 Å	Lu-O$_{water}$= 4.15 Å	Lu-O$_{water}$= 5.00 Å

Figure S8. Minimum energy structures of the A-C isomers with an additional water molecule.

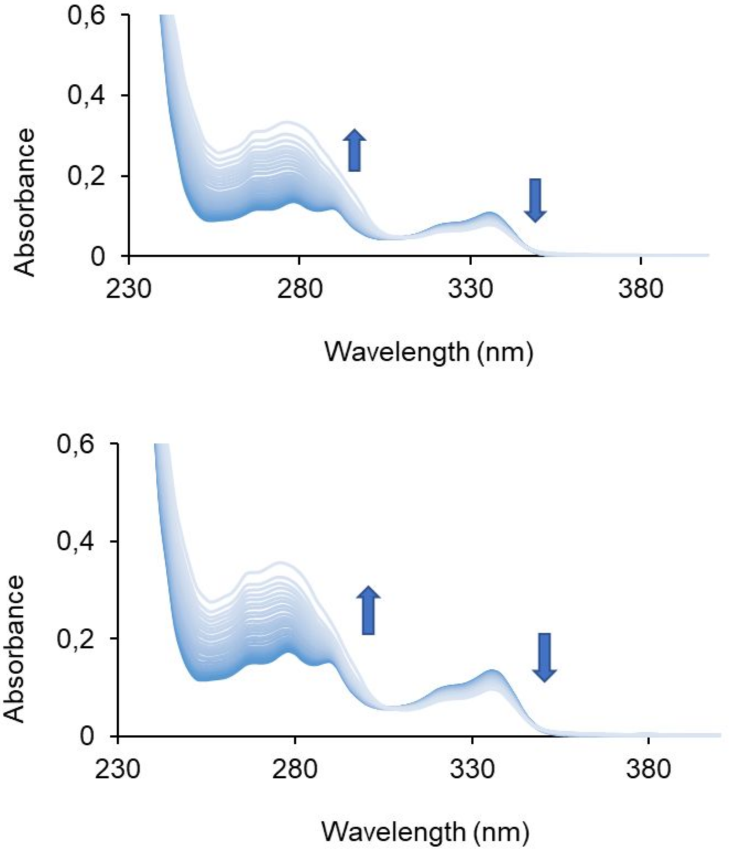

Figure S9. Spectrophotometric titration in dry methanol of (top) *S*-BINOL (16.9 μM) with [Yb(*R,R*)-**L**](NO_3)$_3$ and (bottom) *R*-BINOL (20.8 μM) with [Yb(*R,R*)-**L**](NO_3)$_3$ (71.4 μM). The formation constants were obtained by multi-wavelength analysis of the absorption spectra using HypSpec.[1]

3. Chiroptical spectroscopy

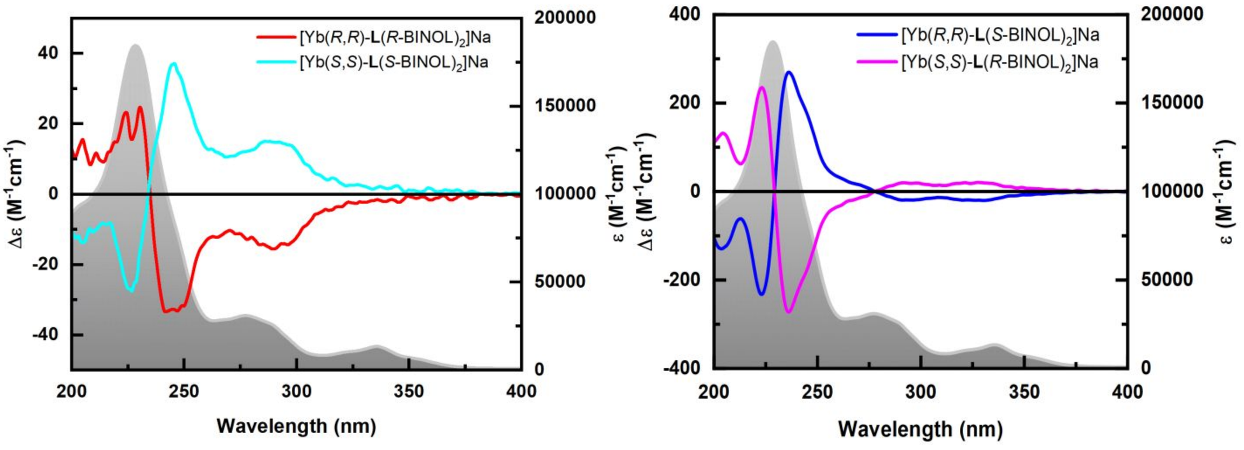

Figure S10. ECD spectra of (left) [Yb(*R,R*)-**L**(*R*-BINOL)$_2$]Na (red) and [Yb(*S,S*)-**L**(*S*-BINOL)$_2$]Na (cyano) and (right) [Yb(*R,R*)-**L**(*S*-BINOL)$_2$]Na (blue) and [Yb(*S,S*)-**L**(*R*-BINOL)$_2$]Na (magenta) with the molecular extinction coefficient traced in the background.

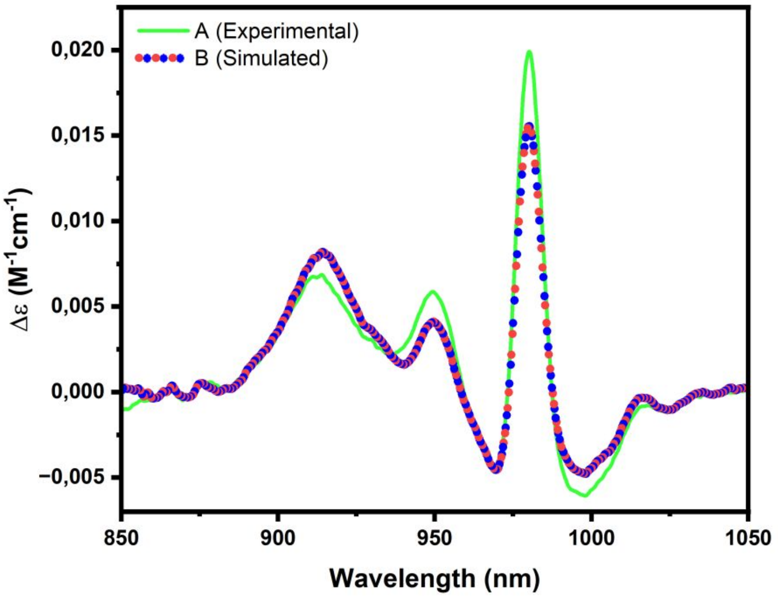

Figure S11. Overlap of A and B where A = Experimental NIR CD of Yb^{3+}/(*R*,*R*)-**L**/(*R*-BINOL)/(*S*-BINOL) 1:1:1:1 mixture (green line) and B = simulated NIR CD average of [Yb(*R,R*)-**L**(*S*-BINOL)$_2$]Na and [Yb(*R,R*)-**L**(*R*-BINOL)$_2$]Na (blue and red dots).

Bibliography

(1) Gans, P.; Sabatini, A.; Vacca, A. Investigation of equilibria in solution. Determination of equilibrium constants with the HYPERQUAD suite of programs. *Talanta* **1996**, *43*, 1739-1753.
